# An evolutionary game theory analysis on the environmental impact of discharging Fukushima’s nuclear wastewater: International stakeholders and strategic dynamics

**DOI:** 10.1371/journal.pone.0317419

**Published:** 2025-01-27

**Authors:** Mingyang Li, Han Pengsihua, Songqing Zhao, Zejun Wang, Limin Yang, Tongjing Liu

**Affiliations:** 1 China University of Petroleum-Beijing at Karamay, Karamay, Xinjiang, China; 2 Changchun Institute of Optics, Fine Mechanics and Physics, Chinese Academy of Sciences, Changchun, Jilin, China; 3 University of Chinese Academy of Sciences, Beijing, China; Universidade de Lisboa Instituto Superior Tecnico, PORTUGAL

## Abstract

On August 24, 2023, Japan controversially decided to discharge nuclear wastewater from the Fukushima Daiichi Nuclear Power Plant into the ocean, initiating intense domestic and global debates. This study employs a mixed-method approach, integrating quantitative evolutionary game theory and qualitative data analysis to explore the strategic dynamics among Japan, other nations, and the Japan Fisheries Association regarding this decision. The data includes international environmental reports and economic statistics, served as the basis for simulating decision-making processes under various legal, economic, and environmental pressures. The evolutionary game theory model is used to predict and analyze three evolutionarily stable strategies (ESS), detailing the transition from the initiation to cessation of wastewater discharge. These strategies highlight the necessity for international cooperation, rigorous scientific research, public education, and effective wastewater treatment methods. This study aims to provide both a theoretical framework and practical guidance to foster a global consensus on nuclear wastewater management, which is vital for marine conservation and sustainable development.

## Introduction

In 2011, the world witnessed the Fukushima disaster, marking the most severe nuclear catastrophe since the Chernobyl disaster, leaving an indelible imprint on human history. In the initial six weeks following the disaster, the amount of radioactive materials released from Fukushima was detected to be substantial, with the release of cesium amounting to 42% of the total release in the Chernobyl disaster, and the release of xenon reaching an unprecedented level [1]. In response to this crisis, the Japanese government adopted water injection measures to slow down the melting speed of the fuel rods. However, this measure led to another crisis—large amounts of nuclear-contaminated water needed to be sealed and stored, posing not only a high cost but also a potential permanent burden (International Atomic Energy Agency, IAEA, 2021) [2]. To address this pressing issue, the Japanese government initiated a series of intense discussions and evaluations, exploring various possible solutions. Among numerous solutions, discharging wastewater into the sea was considered a relatively practical approach, albeit filled with controversy and risk. After a series of careful considerations and weighing of pros and cons, on April 13, 2021, the Japanese government officially decided to discharge 1.2 million tons of Fukushima wastewater into the sea [3], a decision that immediately attracted widespread attention worldwide.

Despite the Japanese government’s repeated assurances that the discharged polluted water had been treated and the radiation values met safety standards, this action still provoked strong opposition from the international community [4], as reflected in the diverse responses captured in media representations across Japan and neighboring countries, discussed in detail by Gong et al. (2024) [5].

On August 24, 2023, the Japanese government officially commenced the discharge of nuclear wastewater from the Fukushima Daiichi Nuclear Power Station into the ocean, once again triggering extensive international attention and controversy [6]. Although the International Atomic Energy Agency (IAEA) deems Japan’s plan to discharge treated water from Fukushima into the sea to be in compliance with international safety standards [7], many countries have expressed strong opposition due to the uncertainty of the potential dangers and long-term impacts brought by such astonishing discharge. Among them, China firmly opposes Japan’s unilateral decision affecting the entire international community and condemns this action [8]. Japan’s nuclear wastewater discharge plan is essentially resolving its own nuclear safety threat by sacrificing the safety of the human marine environment [9]. In this contentious context, Ng Kwan Hoong and colleagues (2022) offer critical perspectives on the possible avenues through which the Japanese government could engage more effectively with the international community to alleviate concerns and build consensus around their wastewater management strategies [10]. As early as the end of 2018, Tokyo Electric Power Company had already admitted that about 80%, i.e., 890,000 tons, of the then stored 1.1 million tons of treated water still contained ^90^Sr, ^60^Co, ^106^Ru, and many other radioactive nuclides exceeding the limit values, and the purification system failed to adequately remove these radioactive nuclides [11]. With the progress of Japan’s nuclear wastewater discharge plan, the world is on the verge of experiencing the most severe nuclear pollution. The seas near Fukushima Prefecture are not only the economic source for coastal residents but also an integral part of the Pacific and the world’s oceans. The substantial amount of radioactive materials will have immeasurable impacts on marine life, the natural environment, and human health.

The act of discharging has already occurred, and the rational handling of Fukushima’s nuclear wastewater is a critical issue that the discharging country and even all stakeholders should focus on. Finding effective approaches is key to resolving governance dilemmas and achieving sustainable development of the oceans. In this context, exploring the long-term relationships between the Japanese government, other countries, and the Japanese Fisheries Association is necessary. Viewing these three parties as stakeholders, the dynamic game among them encompasses the outcomes of handling nuclear contaminated water. We utilize an evolutionary game model to discern the pivotal influencing factors and conduct an in-depth study on the impact of various parameters on the evolutionary process under the condition of Japan refraining from discharging nuclear wastewater, aspiring to contribute to the conservation of the living environment for all of humanity.

In Section 2 of this paper, we review literature pertinent to our study. Section 3 provides a detailed explanation of each parameter used in the model, offering a clear theoretical basis for subsequent analysis and discussion. In Section 4, we propose a set of initial assumptions aimed at more accurately constructing the model of benefits and costs for the three parties—Japan, other countries, and the Fisheries Association. Based on these assumptions, we further develop the replicator dynamics equations in the evolutionary game model to reveal the strategic changes and choices of the parties during the game process. Section 5 delves deeply into the behavioral stability of the three parties under different conditions. We not only analyze various types of asymptotic stability in detail but also explore the necessary conditions and possibilities for achieving such stability, providing important theoretical support for the refinement of the theoretical model and empirical analysis. In Section 6, we validate the feasibility and accuracy of the model through numerical simulations, revealing evolutionary trends under different stable conditions. Specifically, we focus on the scenario where Japan decides to cease ocean discharge, analyzing the impact of key parameters on the evolutionary process under this scenario. This not only provides theoretical references for the three parties to seek optimal strategies in practical problems but also helps in more comprehensively understanding how each party responds to different strategies and environmental variables, thereby affecting the final outcome of the evolutionary game.

In the wake of the 2011 Fukushima disaster, significant attention has been devoted to the containment and mitigation of ongoing environmental threats posed by nuclear wastewater. While considerable literature has focused on the immediate chemical hazards and environmental impacts of such discharges, there remains a substantial gap in understanding the strategic interactions among key stakeholders involved in these critical decisions.

Our research aims to fill this gap by applying evolutionary game theory to explore the strategic dynamics and decision-making processes involved in the discharge of Fukushima’s nuclear wastewater. This approach allows us to identify and analyze the factors influencing these decisions and to propose evolutionarily stable strategies that could lead to more sustainable and acceptable outcomes. By doing so, we contribute a new perspective to the discourse on nuclear disaster management, emphasizing the need for a strategic approach that balances environmental integrity with political and economic realities.

In this study, we utilize key parameters in our evolutionary game theory model to understand the strategic interactions concerning Fukushima’s nuclear wastewater discharge. Each symbol represents a specific variable critical to modeling the decisions and interactions among the Japanese government (*J*), other countries (*C*), and the Japanese Fisheries Association (*F*). These include probabilities such as *x* and *y* which represent Japan’s likelihood to continue or cease discharge and other countries’ decisions to sanction, respectively. Costs like *C*_*DJ*_ (discharge cost) and *C*_*SJ*_ (storage cost) quantify the financial implications of each decision. Additionally, variables like *I*_*J*_ and *C*_*IF*_ reflect the international image repercussions for Japan and the Fisheries Association. Further, *E*_*RF*_, *T*_*RJ*_, and *C*_*SC*_ assess economic impacts from changes in revenue, tax, and seafood production costs. The inclusion of these parameters provides a comprehensive framework to simulate and analyze the outcomes of different strategic scenarios, as detailed in Table 1.

**Table 1:** Description of parameters.

Parameter	Description
*J*	Japanese Government
*C*	Other Countries
*F*	Japanese Fisheries Association
*x*	Probability of Japan choosing the discharge strategy
1 − *x*	Probability of Japan choosing to cease discharge
*y*	Probability of other countries choosing to sanction Japanese discharge
1 − *y*	Probability of other countries choosing not to sanction Japanese discharge
*z*	Probability of the Japanese Fisheries Association opposing the Japanese government
1 − *z*	Probability of the Japanese Fisheries Association supporting the Japanese government
*C*_*DJ*_	Cost of Japanese discharge
*C*_*SJ*_	Cost of storing nuclear wastewater in Japan
*I*_*J*_	International image of Japan
*C*_*IF*_	International image of the Japanese Fisheries Association
*C*_*LF*_	Litigation compensation of the Fisheries Association
*C*_*LC*_	Litigation compensation of other countries
*E*_*RF*_	Reduction in revenue for the Fisheries Association due to discharge
*T*_*RJ*_	Reduction in export tax revenue for Japan due to discharge
*C*_*SC*_	Additional cost for other countries to develop their own seafood products
*B*_*SP*_	Potential benefits to other countries’ seafood industries from introducing Japanese seafood substitutes
*C*_*MJ*_	Ocean monitoring cost for the Japanese government in the case of discharge
*C*_*MC*_	Ocean monitoring cost for other countries in the case of discharge
*C*_*HJ*_	Aid received by Japan from other countries in the case of no discharge

## Literature review

### Chemical hazards of Japan’s nuclear wastewater discharge into the sea

On March 11, 2011, a severe earthquake occurred in the northeastern sea area of Japan, triggering a tsunami and causing serious damage to the Fukushima Daiichi Nuclear Power Plant (FDNPP). The failure of the cooling system led to a temperature rise in the reactor, generating hydrogen and other gases, causing explosions and releasing radioactive gases and fragments into the atmosphere. Simultaneously, untreated cooling water was directly discharged into the northeastern sea area of Japan, making this incident one of the most severe uncontrolled inputs of artificial radionuclides into the ocean in history [12]. According to the water treatment facility monitoring reports by Tokyo Electric Power Company Holdings (TEPCO, 2012–2020) and the seawater quality monitoring reports by the Nuclear Regulation Authority (NRA, 2013–2020), the radioactive substances mainly include ^3^H, ^14^C, ^134^Cs, ^137^Cs, ^60^Co, ^125^Sb, ^90^Sr, ^129^I, ^99^Tc, ^106^Ru, and ^238^Pu [13]. Although the concentrations of these isotopes are lower than tritium, they are more likely to integrate into marine organisms and seabed sediments (Buesseler, 2020) [14]. If nuclear wastewater is discharged into the Pacific Ocean, potential risks will exist for hundreds or even thousands of years in the future. The marine food web tightly connects marine organisms through predation (Albouy et al., 2019) [15]. Cesium isotopes are the main pollutants in the accumulated seawater inside Japanese nuclear facilities and tanks. Additionally, cesium is a highly soluble radioactive nuclide in seawater, posing long-term radiation risks to the environment and is more likely to integrate into marine organisms or sediments under high concentration factors (Buesseler, 2020) [14]. The harm of cesium to humans is mainly reflected in its radioactive radiation and human absorption. Moreover, the concentration of ^14^C in treated nuclear wastewater is also high (the Korea Times, 2021b) [16], it releases low-energy *β* particles and may enter the biosphere and accumulate in marine ecosystems (Williams et al, 2010) [17]. Co is also a radioactive isotope that releases gamma rays, which can penetrate the human body and cause cell damage (Khajeh et al, 2017) [18]. ^90^Sr can simulate calcium in the human body, significantly increasing the risk of osteosarcoma and leukemia (Khani et al., 2012) [19]. The ocean has a large volume and a complex current system, with strong abilities to dilute and disperse radioactive substances, but long half-life radioactive nuclides will still exist in the marine environment for a long time (Men and Deng et al., 2017) [20], causing significant consequences to the natural environment, marine life, and human health.

### Evolutionary game theory

Game theory was originally designed to analyze economic behavior and, with modifications, can also be applied to evolving populations. The concept of “Evolutionarily Stable Strategy” (ESS) proposed by Smith (1982) reveals the relevance of the optimal behavior of animals or plants under the influence of the behavior of other individuals [21]. In classical game theory, the basic components of a game include players, strategies, and payoff matrices, and the results of the game can be clearly displayed through the payoff matrices. Evolutionary game theory, originating from classical game theory, has gradually expanded its range of application.

Smith (1973) regarded evolutionary game theory as a theoretical framework for studying the interaction between individual behavior and adaptation. According to this theory, the evolutionary process is a dynamic strategy selection process, where the fitness of an individual depends not only on the chosen strategy but also on interactions with other individuals [22]. Adami et al. (2016) believed that the main goal in the application of evolutionary game theory is to find suitable strategies or optimal decision sequences to resolve existing conflicts and obtain maximum benefits. However, the competition between different strategies does not occur simultaneously at the same point in time but changes over specific periods. New strategies continuously emerge, compete with existing ones, and may replace the originally dominant strategies by gaining advantages in space and time. Therefore, the success of a strategy depends on its competition with other strategies during a specific period [23]. Evolutionary game models are important tools for exploring social behavior. Predecessors have constructed various types of evolutionary game models through extensive research to explain the emergence and continuation of various social behaviors. It has been widely used in economics, sociology, and statistical physics to analyze various factors affecting the formation of group behavior, producing rich and influential research results (Perc and Jordan et al., 2017; Khoo and Fu et al., 2018; Wang and He et al., 2018)[24–27].

Notably, ESS (Evolutionarily Stable Strategy) is a powerful tool in game theory to find optimal strategies that can prevail in the competition with other strategies [28].

### Application of evolutionary game theory in this study

This global concern has spurred significant research into the containment and mitigation of ongoing environmental threats posed by such nuclear wastewater. Despite extensive discussions on the immediate chemical hazards and environmental impacts of these discharges, there remains an evident gap in the literature regarding the strategic interactions among the key stakeholders managing these critical decisions. Following Japan’s decision to discharge nuclear wastewater into the sea, researchers have begun to employ evolutionary game theory to study this issue.

Zheng (2022) constructed an evolutionary game model involving fishermen, consumers, and the government, analyzing the impact of each entity’s behavior probability on the strategies of other entities and the stability of the entire system. However, this study did not consider the impact of international cooperation and conflicts on strategy evolution [29]. Xin et al. (2022) explored an evolutionary game considering emotional impacts to study the evolutionarily stable strategies of the Japanese government and Japanese fishermen. This study primarily focused on domestic factors and did not fully consider the dynamics and interactions at the international level [30]. Xu et al. (2022) established a tripartite evolutionary game model including the International Atomic Energy Agency (IAEA), the Discharging Country (DC), and the Fisheries Cooperative Association of Japan (FCA), aiming to propose management insights for international cooperation. However, this study did not fully explore the changes and evolution of strategies among the parties in the game, an aspect this paper intends to analyze more comprehensively [31]. Su et al. (2023) constructed a Tripartite Evolutionary Game (TEG) model to analyze the evolutionary stability of strategy choices among fishermen, cooperatives, and government departments, exploring influencing factors and their relationships. The results indicate that the profits of cooperatives and the cooperation costs of fishermen are key factors affecting the strategy choices of the three parties in the game. However, this study did not consider the impact of international factors on strategy choices [32]. Zhang et al. (2023) adopted a complex network evolutionary game based on the WS small-world model, focusing on the fisherman population, to explore the dynamic evolutionary laws of cooperative behavior diffusion in the development of marine carbon sink fisheries. This study did not involve the roles and strategies of other countries and international organizations, an aspect that is supplemented and expanded upon in this paper [33]. Previous research on Japan’s nuclear wastewater discharge was predominantly predictive, given that Japan only commenced the actual discharge on August 24, 2023. Consequently, studies prior to this date were often based on limited information and considerations. In contrast, our research, informed by post-discharge realities, incorporates a broader spectrum of practical factors and employs evolutionary game theory to delve into the strategic decisions and interactions among the stakeholders. Our approach amalgamates perspectives from economics, environmental science, and international relations, with a particular emphasis on real-time international impacts and responses—elements frequently overlooked in earlier studies. Notably, our work pioneers the exploration of the balance of interests among the Japanese government, other nations, and the Japan Fisheries Association. Furthermore, we are the first to apply evolutionary game theory within this specific context. Our study not only broadens the knowledge frontier of evolutionary game models in environmental policy and international relations but also, through a tripartite framework, adeptly addresses the literature gap concerning the tripartite balance of interests in Japan’s marine discharge policy. In essence, our analysis, rooted in an international dimension, offers a comprehensive and realistic perspective on the dynamic games surrounding Japan’s nuclear wastewater issue.

## Symbol definitions

All symbols and their corresponding meanings are detailed and illustrated in Table 1.

## Methodology

### Basic assumptions

1. In this tripartite game, Japan (J), the National Fisheries Association of Japan (F), and other countries (C) are set as agents. Due to limited information and rationality, the decisions of each party are constrained.
2. Employing the method of evolutionary game theory, the aforementioned three parties are considered as stakeholders. The dynamic game among these stakeholders involves their respective pursuits of interest and choices of action.
3. In this game, Japan (J), as the country discharging nuclear-contaminated water, might weigh domestic economic interests against international reputation, deciding whether to continue the discharge of nuclear-contaminated water. The probability of discharging is denoted as *x*, and conversely, as 1 − *x*.
4. Other countries (C), as additional stakeholders, may exhibit attitudes of either sanctioning or not sanctioning. Some countries might express dissatisfaction with Japan’s discharge strategy and adopt sanction measures with a probability of *y*, to protect international environmental and marine resource interests. Conversely, some might choose not to sanction, with a probability of 1 − *y*.
5. The National Fisheries Association of Japan (F) represents the interests of Japanese fisheries. Their decisions may manifest in two forms: one opposing Japan’s discharge strategy with a probability of *z*, perceiving it as detrimental to fishery resources; the other, with a probability of 1 − *z*, might not oppose and thus would endorse Japan’s discharge actions.
6. This study solely analyzes the game strategies of each party from an economic perspective and does not consider the impact of political and other factors on the strategic choices of each participant.

**Fig 1:**
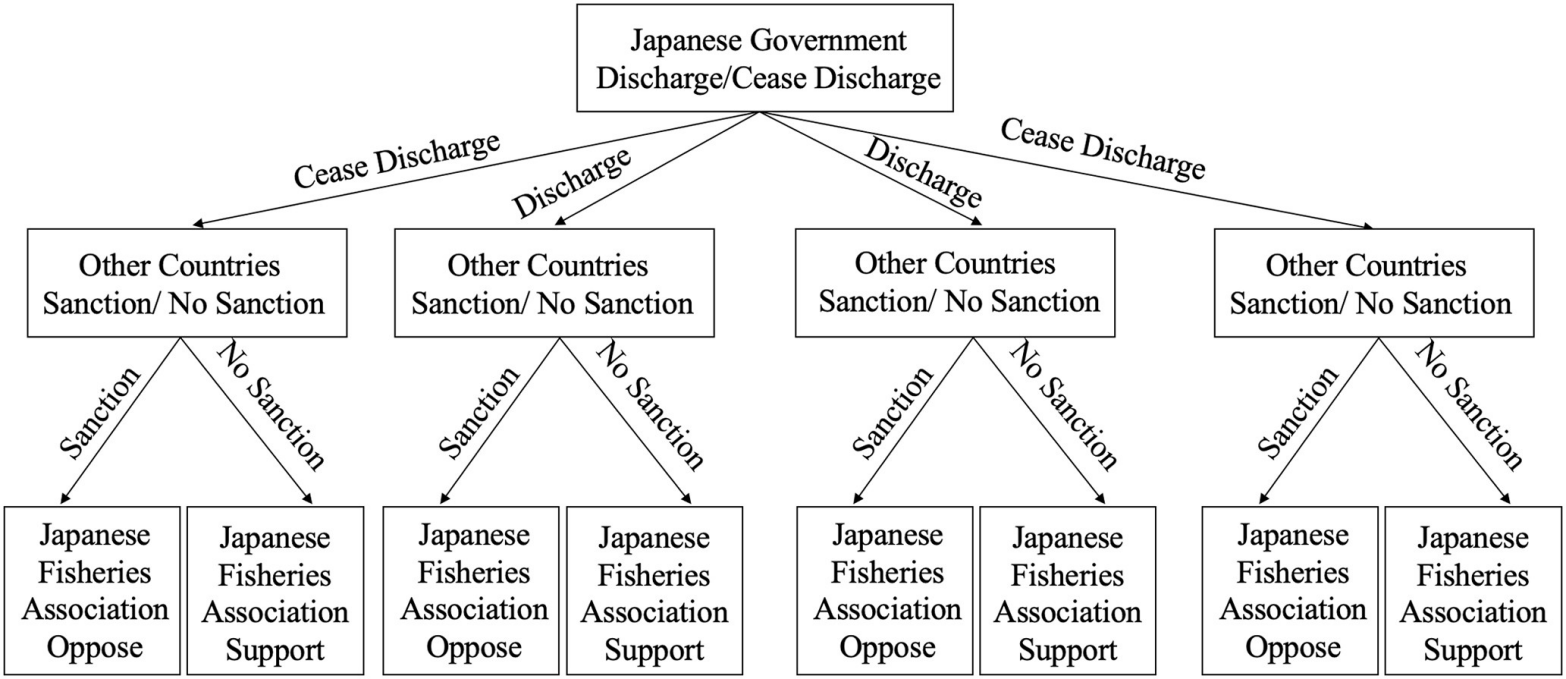


Each participant’s occurrence in all possible scenarios under different strategies is illustrated in Fig 1.

### Evolutionary game model

The payoff matrix for the Japanese government, other countries, and the Japanese Fisheries Association is shown in Table 2.

**Table 2:** Payoff matrix of Japan, other countries, and japanese fisheries association.

Stakeholders				Japanese Fisheries Association	
				Opposition (*z*)	Acceptance (1 − *z*)
**Japan**	Discharge (*x*)	**Other Countries**	Sanction (*y*)	− *I*_*J*_ − *C*_*LF*_ − *C*_*LC*_ − *T*_*RJ*_ − *C*_*DJ*_ − *C*_*MJ*_, − *C*_*SC*_ + *B*_*SP*_ + *C*_*LC*_ − *C*_*MC*_, *C*_*LF*_ − *E*_*RF*_	>− *I*_*J*_ − *C*_*LC*_ − *T*_*RJ*_ − *C*_*DJ*_ − *C*_*MJ*_, − *C*_*SC*_ + *B*_*SP*_ + *C*_*LC*_ − *C*_*MC*_, *E*_*RF*_ − *C*_*IF*_
			No Sanction (1 − *y*)	− *C*_*LF*_ − *C*_*DJ*_ − *C*_*MJ*_, − *C*_*MC*_, *C*_*LF*_	− *C*_*DJ*_ − *C*_*MJ*_, − *C*_*MC*_, *C*_*IF*_
	No Discharge (1 − *x*)		Sanction (*y*)	*C*_*HJ*_ − *C*_*SJ*_, − *C*_*HJ*_, 0	*C*_*HJ*_ − *C*_*SJ*_, − *C*_*HJ*_, − *C*_*IF*_
			No Sanction (1 − *y*)	− *C*_*SJ*_, 0, 0	− *C*_*SJ*_, 0, − *C*_*IF*_

Assume that the utility of the Japanese government choosing to discharge nuclear wastewater is denoted as *U*_11_, which encapsulates the benefits and costs associated with this action, including economic gains and potential international backlash. Conversely, the utility of the Japanese government deciding against discharging is denoted as *U*_12_. This reflects the potential benefits from maintaining environmental standards and avoiding international sanctions, minus any internal costs of managing stored wastewater. The average expected utility for Japan, taking into account the probability of each strategy being employed, is then represented by ${\bar{U}}_{1}$. The formula for ${\bar{U}}_{1}$ is given by:

$$\begin{array}{c} {\bar{U}}_{1}=x\cdot U_{11}+\left(1-x\right)\cdot U_{12} \end{array}$$ (1)

where *x* is the probability of choosing to discharge.

$$\begin{array}{cc} U_{11} & =yz(-I_{J}-C_{LF}-C_{LC}-T_{RJ}-C_{DJ}-C_{MJ})\\ & +y\left(1-z\right)\left(-I_{J}-C_{LC}-T_{RJ}-C_{DJ}-C_{MJ}\right)\\ & +\left(1-y\right)z\left(-C_{LF}-C_{DJ}-C_{MJ}\right)+\left(1-y\right)\left(1-z\right)\left(-C_{DJ}-C_{MJ}\right)\\ & =y\left(-I_{J}-C_{LC}-T_{RJ}\right)-zC_{LF}-C_{DJ}-C_{MJ} \end{array}$$ (2)

$$\begin{array}{cc} & U_{12}=yz\left(C_{HJ}-C_{SJ}\right)+y\left(1-z\right)\left(C_{HJ}-C_{SJ}\right)\\ & +\left(1-y\right)z\left(-C_{SJ}\right)+\left(1-y\right)\left(1-z\right)\left(-C_{SJ}\right)\\ & =yC_{HJ}-C_{SJ} \end{array}$$ (3)

The replicator dynamic equation for the Japanese government, denoted as *S*(*x*), is:

$$\begin{array}{cc} S\left(x\right)=\frac{dx}{dt} & =x\left(U_{11}-{\bar{U}}_{1}\right)=x\left(1-x\right)\left(U_{11}-U_{12}\right)\\ & =x\left(1-x\right)[y\left(-I_{J}-C_{LC}-T_{RJ}-C_{HJ}\right)-zC_{LF}-C_{DJ}-C_{MJ}+C_{SJ}] \end{array}$$ (4)

For other countries, the expected utility of sanctioning the Japanese government is denoted as *U*_21_. This utility reflects the benefits derived from imposing sanctions aimed at enforcing international environmental norms, potentially including improved global environmental outcomes and international prestige, minus the costs of enforcing these sanctions. The utility of not sanctioning is denoted as *U*_22_, which could include maintaining trade relations with Japan or avoiding the diplomatic costs of sanctioning. The average expected utility for other countries, reflecting their collective strategy towards Japan, is represented by ${\bar{U}}_{2}$. It is calculated as follows:

$$\begin{array}{c} {\bar{U}}_{2}=yU_{21}+\left(1-y\right)U_{22} \end{array}$$ (5)

where *y* is the probability of sanctioning Japan.

$$\begin{array}{cc} & U_{21}=xz\left(-C_{SC}+B_{SP}+C_{LC}-C_{MC}\right)+x\left(1-z\right)\left(-C_{SC}+B_{SP}+C_{LC}-C_{MC}\right)\\ & +\left(1-x\right)z\left(-C_{HJ}\right)+\left(1-x\right)\left(1-z\right)\left(-C_{HJ}\right)\\ & =x\left(C_{HJ}-C_{SC}+B_{SP}+C_{LC}-C_{MC}\right)-C_{HJ} \end{array}$$ (6)

$$\begin{array}{cc} & U_{22}=xz\left(-C_{MC}\right)+x\left(1-z\right)\left(-C_{MC}\right)+\left(1-x\right)z\cdot 0+\left(1-x\right)\left(1-z\right)\cdot 0\\ & =-xC_{MC} \end{array}$$ (7)

The replicator dynamic equation for other countries, denoted as *G*(*y*), is:

$$\begin{array}{cc} G\left(y\right)=\frac{dy}{dt} & =y\left(U_{21}-{\bar{U}}_{2}\right)=y\left(1-y\right)\left(U_{21}-U_{22}\right)\\ & =y\left(1-y\right)[x\left(C_{HJ}-C_{SC}+B_{SP}+C_{LC}\right)-C_{HJ}] \end{array}$$ (8)

Assume that the utility of the Japan Fisheries Association opposing the discharge of nuclear wastewater is represented by *U*_31_. This utility might include the benefits of preserving marine life and fisheries resources, which are critical to their economic interests, offset by any potential conflicts with government policies or economic repercussions from reduced national industrial activities. Conversely, the utility of the Fisheries Association supporting the discharge is denoted as *U*_32_. This might reflect the potential benefits of aligning with government policies or economic gains from continued industrial operations, minus the environmental costs and potential long-term damage to fisheries.

The average expected utility for the Japan Fisheries Association, which considers the probabilities of opposing or supporting the discharge, is calculated as:

$$\begin{array}{c} {\bar{U}}_{3}=zU_{31}+\left(1-z\right)U_{32} \end{array}$$ (9)

where *z* is the probability of the Association opposing the discharge.

$$\begin{array}{cc} & U_{31}=xy\left(C_{LF}-E_{RF}\right)+x\left(1-y\right)\left(C_{LF}\right)+\left(1-x\right)y\cdot 0+\left(1-x\right)\left(1-y\right)\cdot 0\\ & =-xyE_{RF}+xC_{LF} \end{array}$$ (10)

$$\begin{array}{cc} & U_{32}=xy\left(-E_{RF}-C_{IF}\right)+x\left(1-y\right)\left(-C_{IF}\right)+\left(1-x\right)y\left(-C_{IF}\right)+\left(1-x\right)\left(1-y\right)\left(-C_{IF}\right)\\ & =-xyE_{RF}-C_{IF} \end{array}$$ (11)

$$\begin{array}{c} {\bar{U}}_{3}=zU_{31}+\left(1-z\right)U_{32} \end{array}$$ (12)

The replicator dynamic equation for the Japan Fisheries Association, denoted as *P*(*z*), is:

$$\begin{array}{cc} P\left(z\right)=\frac{dz}{dt} & =z\left(U_{31}-{\bar{U}}_{3}\right)=z\left(1-z\right)\left(U_{31}-U_{32}\right)\\ & =z\left(1-z\right)\left(xC_{LF}+C_{IF}\right) \end{array}$$ (13)

To further discuss the stable points in evolutionary game theory, we combine the Eqs (4), (8) and (13) to obtain the system of replicator dynamic equations as follows:

$$\begin{array}{c} \left\{ \begin{array}{c} S\left(x\right)=\frac{dx}{dt}=x\left(1-x\right)[y\left(-I_{j}-C_{LC}-T_{RJ}-C_{HJ}\right)-zC_{LF}-C_{DJ}-C_{MJ}+C_{SJ}]\\ G\left(y\right)=\frac{dy}{dt}=y\left(1-y\right)\left[x\left(C_{HJ}-C_{SC}+B_{SP}+C_{LC}\right)-C_{HJ}\right]\\ P\left(z\right)=\frac{dz}{dt}=z\left(1-z\right)\left(xC_{LF}+C_{IF}\right) \end{array}\right. \end{array}$$ (14)

## Evolutionary stability analysis

### Asymptotic stability analysis of the three parties

Let the dynamic replicator equations (14) be defined as *S*(*x*) = *G*(*y*) = *P*(*z*) = 0. By solving these, we can identify eight pure strategy equilibria and one mixed strategy equilibrium, which are *γ*_1_(0, 0, 0)^*T*^, *γ*_2_(1, 0, 0)^*T*^, *γ*_3_(0, 1, 0)^*T*^, *γ*_4_(0, 0, 1)^*T*^, *γ*_5_(1, 1, 0)^*T*^, *γ*_6_(1, 0, 1)^*T*^, *γ*_7_(0, 1, 1)^*T*^, *γ*_8_(1, 1, 1)^*T*^, and *γ*_9_(x*, y*, z*)^*T*^, where *γ*_9_(x*, y*, z*)^*T*^ is determined by the following equations:

$$\begin{array}{c} \left\{ \begin{array}{c} y^{*}\left(-I_{j}-C_{LC}-T_{RJ}-C_{HJ}\right)-z^{*}C_{LF}-C_{DJ}-C_{MJ}+C_{SJ}=0\\ x^{*}\left(C_{HJ}-C_{SC}+B_{SP}+C_{LC}\right)-C_{HJ}=0\\ x^{*}C_{LF}+C_{IF}=0 \end{array}\right. \end{array}$$ (15)

In asymmetric games, Evolutionarily Stable Strategies (ESS) are pure strategy equilibria, hence we only consider the asymptotic stability of pure strategies. The stability of equilibrium points in evolutionary games is typically determined by the stability of the Jacobian matrix, as illustrated below.

$$\begin{array}{c} J=[\begin{array}{ccc} J_{11} & J_{12} & J_{13}\\ J_{21} & J_{22} & J_{23}\\ J_{31} & J_{32} & J_{33} \end{array}]=[\begin{array}{ccc} \frac{\partial S(x)}{\partial x} & \frac{\partial S(x)}{\partial y} & \frac{\partial S(x)}{\partial z}\\ \frac{\partial G(y)}{\partial x} & \frac{\partial G(y)}{\partial y} & \frac{\partial G(y)}{\partial z}\\ \frac{\partial P(z)}{\partial x} & \frac{\partial P(z)}{\partial y} & \frac{\partial P(z)}{\partial z} \end{array}] \end{array}$$ (16)

where

$$\begin{array}{c} J_{11}=\left(1-2x\right)[y\left(-I_{j}-C_{LC}-T_{RJ}-C_{HJ}\right)-zC_{LF}-C_{DJ}-C_{MJ}+C_{SJ}] \end{array}$$ (17)

$$\begin{array}{c} J_{12}=x\left(1-x\right)\left(-I_{j}-C_{LC}-T_{RJ}-C_{HJ}\right) \end{array}$$ (18)

$$\begin{array}{c} J_{13}=x\left(1-x\right)\left(-C_{LF}\right) \end{array}$$ (19)

$$\begin{array}{c} J_{21}=y\left(1-y\right)\left(C_{HJ}-C_{SC}+B_{SP}+C_{LC}\right) \end{array}$$ (20)

$$\begin{array}{c} J_{22}=\left(1-2y\right)\left[x\left(C_{HJ}-C_{SC}+B_{SP}+C_{LC}\right)-C_{HJ}\right] \end{array}$$ (21)

$$\begin{array}{c} J_{23}=0 \end{array}$$ (22)

$$\begin{array}{c} J_{31}=z\left(1-z\right)C_{LF} \end{array}$$ (23)

$$\begin{array}{c} J_{32}=0 \end{array}$$ (24)

$$\begin{array}{c} J_{33}=\left(1-2z\right)\left(xC_{LF}+C_{IF}\right) \end{array}$$ (25)

According to Lyapunov stability theory, the stability of equilibrium points in evolutionary games can be determined by the eigenvalues of the Jacobian matrix [34]. An equilibrium point is asymptotically stable, and the strategy is an Evolutionarily Stable Strategy (ESS), only if all the eigenvalues of the Jacobian matrix are negative.

By analyzing the computed results for each point (in Appendix), the stability analysis of these points can be found as shown in Table 3.

**Table 3:** Stability analysis of equilibrium point.

Equilibrium Point	Eigenvalues		Stability	Condition
	λ(λ_1_, λ_2_, λ_3_)	Symbol		
*γ*_1_(0, 0, 0)^*T*^	*C*_*SJ*_ − *C*_*MJ*_ − *C*_*DJ*_ − *C*_*HJ*_ *C*_*IF*_	(*,-, +)	Non-ESS	
*γ*_2_(1, 0, 0)^*T*^	*C*_*DJ*_ + *C*_*MJ*_ − *C*_*SJ*_ *B*_*SP*_ + *C*_*LC*_ − *C*_*SC*_ *C*_*LF*_ + *C*_*IF*_	(*,*, +)	Non-ESS	
*γ*_3_(0, 1, 0)^*T*^	*C*_*SJ*_ − *C*_*HJ*_ − *C*_*LC*_ − *C*_*MJ*_ − *C*_*DJ*_ − *I*_*J*_ − *T*_*RJ*_ *C*_*HJ*_ *C*_*IF*_	(*,+, +)	Non-ESS	
*γ*_4_(0, 0, 1)^*T*^	*C*_*SJ*_ − *C*_*LF*_ − *C*_*MJ*_ − *C*_*DJ*_ − *C*_*HJ*_ − *C*_*IF*_	(-,-, -)	ESS	*C*_*SJ*_ < (*C*_*LF*_ + *C*_*MJ*_ + *C*_*DJ*_)
*γ*_5_(1, 1, 0)^*T*^	*C*_*DJ*_ + *C*_*HJ*_ + *C*_*LC*_ + *C*_*MJ*_ − *C*_*SJ*_ + *I*_*J*_ + *T*_*RJ*_ *C*_*SC*_ − *C*_*LC*_ − *B*_*SP*_ *C*_*LF*_ + *C*_*IF*_	(*,*, +)	Non-ESS	
*γ*_6_(1, 0, 1)^*T*^	*C*_*DJ*_ + *C*_*LF*_ + *C*_*MJ*_ − *C*_*SJ*_ *B*_*SP*_ + *C*_*LC*_ − *C*_*SC*_ − *C*_*IF*_ − *C*_*LF*_	(-,-,-)	ESS	*C*_*SJ*_ > (*C*_*LF*_ + *C*_*MJ*_ + *C*_*DJ*_) *B*_*SP*_ + *C*_*LC*_ < *C*_*SC*_
*γ*_7_(0, 1, 1)^*T*^	*C*_*SJ*_ − *C*_*HJ*_ − *C*_*LC*_ − *C*_*LF*_ − *C*_*MJ*_ − *C*_*DJ*_ − *I*_*J*_ − *T*_*RJ*_ *C*_*HJ*_ − *C*_*IF*_	(*,+,-)	Non-ESS	
*γ*_8_(1, 1, 1)^*T*^	*C*_*DJ*_ + *C*_*HJ*_ + *C*_*LC*_ + *C*_*LF*_ + *C*_*MJ*_ − *C*_*SJ*_ + *I*_*J*_ + *T*_*RJ*_ *C*_*SC*_ − *C*_*LC*_ − *B*_*SP*_ − *C*_*IF*_ − *C*_*LF*_	(-,-, -)	ESS	*C*_*SJ*_ > *C*_*DJ*_ + *C*_*HJ*_ + *C*_*LC*_ + *C*_*LF*_ + *C*_*MJ*_ + *I*_*J*_ + *T*_*RJ*_ *C*_*SC*_ < *C*_*LC*_ + *B*_*SP*_

* Note: “-” indicates that the eigenvalue of the Jacobian matrix is negative, “+” means that the eigenvalue is positive, and “*” indicates that the sign of the eigenvalue is uncertain.

Table 3 shows that the equilibrium points *γ*_1_(0, 0, 0)^*T*^, *γ*_2_(1, 0, 0)^*T*^, *γ*_3_(0, 1, 0)^*T*^, *γ*_5_(1, 1, 0)^*T*^ and *γ*_7_(0, 1, 1)^*T*^ all have positive eigenvalues, hence they are unstable points. The equilibrium points *γ*_4_(0, 0, 1)^*T*^, *γ*_6_(1, 0, 1)^*T*^ and *γ*_8_(1, 1, 1)^*T*^ can all have negative eigenvalues. Therefore, they are stable points.

- When C_SJ_ < (C_LF_ + C_MJ_ + C_DJ_), the eigenvalues of the Jacobian matrix corresponding to the evolutionary stable point *γ*_4_(0, 0, 1)^*T*^ are all negative, indicating that *γ*_4_(0, 0, 1)^*T*^ is an evolutionarily stable point. Under this condition, when the cost for Japan to store nuclear wastewater is less than the sum of the costs of discharging it into the sea, marine monitoring fees, and litigation compensation to the Fisheries Association, Japan is more likely to choose the “non-discharge” strategy. By adopting this strategy, Japan can reduce the direct impact on the marine ecosystem, respond to public concerns, protect the image and reputation of the government, potentially gain a competitive advantage in the field of environmental protection technology, and promote international cooperation. Other countries tend to choose the “non-sanction” strategy, as they might worry that sanctioning Japan when it chooses “non-discharge” could have negative impacts on bilateral trade, investment, and economic cooperation, thereby damaging their own economic interests, leading them to choose a more conservative strategy. For the Japanese Fisheries Association, it tends to choose the “oppose” strategy because, even under this condition where the cost of storing nuclear wastewater is less than other related costs, storing nuclear wastewater cannot completely eliminate the potential risks to the marine environment. The association needs to consider that such risks might cause long-term damage to the marine ecosystem and fishery resources, bringing uncertainty to fishing activities and the livelihoods of fishermen.
- When *C*_*SJ*_ > (*C*_*LF*_ + *C*_*MJ*_ + *C*_*DJ*_) and *B*_*SP*_ + *C*_*LC*_ < *C*_*SC*_, the eigenvalues of the Jacobian matrix corresponding to the evolutionary stable point *γ*_6_(1, 0, 1)^*T*^ are all negative, indicating that *γ*_6_(1, 0, 1)^*T*^ is an evolutionarily stable point. At this time, the cost for Japan to store nuclear wastewater exceeds the sum of the costs of discharging it into the sea, marine monitoring fees, and litigation compensation to the Japanese Fisheries Association; the potential benefits and litigation compensation obtained by other countries from introducing substitutes for Japanese seafood are less than the additional costs for other countries to develop their own seafood products. In this scenario, Japan is more likely to choose the “discharge” strategy, allowing it to reduce the costs associated with storing nuclear wastewater. Other countries are more inclined to choose the “non-sanction Japan” strategy, as they can fill the domestic supply gap of Japanese seafood by introducing substitutes and increase their own seafood exports, hence they might prefer introducing seafood substitutes over pursuing litigation compensation. The Japanese Fisheries Association may choose to “oppose” due to a sharp decline in seafood income caused by marine pollution.
- When C_SJ_ > (C_DJ_ + C_HJ_ + C_LC_ + C_LF_ + C_MJ_ + I_J_ + T_RJ_) and C_SC_ < C_LC_ + B_SP_, all the eigenvalues of the Jacobian matrix corresponding to the evolutionary stable point *γ*_8_(1, 1, 1)^*T*^ are negative. This denotes that *γ*_8_(1, 1, 1)^*T*^ is an evolutionarily stable point. Under such circumstances, the cost incurred by Japan to store nuclear wastewater surpasses the aggregate of the costs related to sea discharge, acquiring aid from other nations, compensations from litigations to other nations and the domestic Fisheries Association, marine surveillance fees, impacts on Japan’s global image, and the decrement in export tax revenue due to sea discharge. Concurrently, the supplementary cost for other nations to cultivate their seafood is inferior to the aggregate of the litigation compensations and the prospective gains to their seafood industry from adopting Japanese seafood substitutes. In this context, Japan is inclined to opt for the “discharge” strategy, mitigating the expenses linked to nuclear wastewater storage. The Japanese Fisheries Association is predisposed to “oppose,” as its cardinal objective is to safeguard and advance the domestic fisheries sector’s interests. The discharge of nuclear wastewater into the sea could inflict irreversible damages to the marine ecosystem and pose potential hazards to fisheries resources and the fishing sector. This discharge may contaminate fisheries resources and compromise the quality of the catches, adversely impacting the revenue and living conditions of the fishermen. When the additional cost for other nations to cultivate their seafood is less than the combined litigation compensations and the prospective advantages to their seafood industry from adopting Japanese seafood substitutes, other nations are likely to select the “sanction” strategy. This choice aims to preserve the interests of their seafood sector, secure economic gains from litigation compensations, and sustain a commendable international image and reputation.

## Numerical simulation

### Numerical simulation of different stable points

In the aforementioned tripartite evolutionary game model, the strategy equilibrium of each game participant is influenced by the strategy choices of other participants. To more intuitively explore the strategy selection process of the three stable equilibrium points mentioned above, we set different parameter values according to different scenarios for numerical simulation to analyze the evolutionary trajectories of stable strategies of the Japanese government, other countries, and the Japanese Fisheries Association.

As can be seen from Table 3, the stable points are *γ*_4_(0, 0, 1)^*T*^, *γ*_6_(1, 0, 1)^*T*^, and *γ*_8_(1, 1, 1)^*T*^. To delve deeper into the dynamic characteristics and evolutionary processes of these stable points, we simulate the evolutionary trajectories of these three stable points. We have set a group of parameter values that meet the stable conditions for each stable point. These parameter values will be used to simulate the evolutionary trajectories of these stable points to understand their dynamic behavior more accurately. The specific parameter values are shown in Table 4, with the initial points all set to [0.5, 0.5, 0.5].

**Table 4:** Basic parameter settings for three stable equilibrium points.

Condition	Stable point	*I*_*J*_	*C*_*LC*_	*T*_*RJ*_	*C*_*HJ*_	*C*_*LF*_	*C*_*DJ*_	*C*_*MJ*_	*C*_*SJ*_	*C*_*IF*_	*B*_*SP*_	*C*_*SC*_
Condition 1	*γ*_4_(0, 0, 1)^*T*^	20	8	5	10	35	3	6	30	1	1	30
Condition 2	*γ*_6_(1, 0, 1)^*T*^	20	8	5	10	20	3	6	30	1	1	30
Condition 3	*γ*_8_(1, 1, 1)^*T*^	20	10	5	10	5	3	6	80	1	1	10

#### Evolutionary trajectory under Condition 1

**Fig 2:**
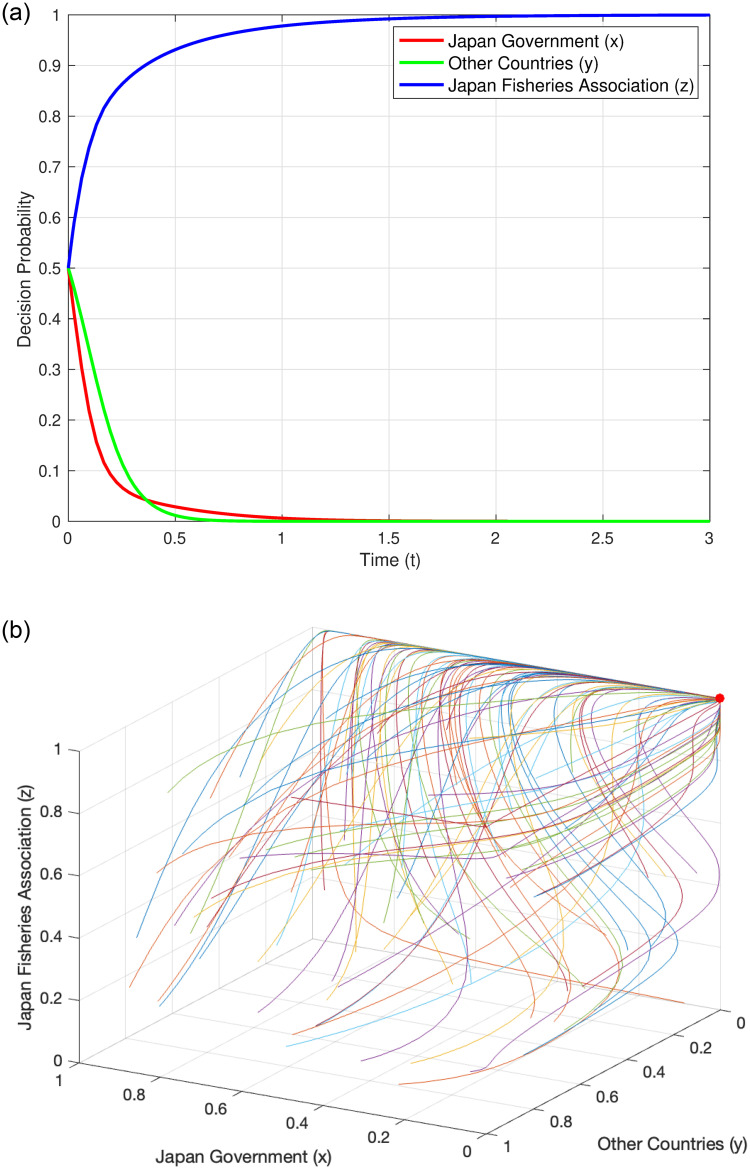


When the system satisfies Condition 1, i.e., C_SJ_ < (C_LF_ + C_MJ_ + C_DJ_), we refer to the parameters of Condition 1 in Table 4 for analysis. This condition implies that when the cost for Japan to store nuclear wastewater is less than the total of other related costs (these costs include the cost of discharging into the sea, marine monitoring fees, and litigation compensation to the Japanese domestic Fisheries Association), the Japanese government is more likely to choose the “non-discharge” strategy. This is because, under such circumstances, choosing to store nuclear wastewater instead of discharging it into the sea is more economical for Japan from a financial perspective. The results of numerical simulation further confirm this viewpoint. The simulation results show that under this condition, the stable point of the system is (0, 0, 1). Fig 2a displays the evolution of the probability of each decision-maker over time, clearly showing how each entity gradually tends towards this stable point over time. Fig 2b illustrates the overall evolutionary trajectory under Condition 1, providing us with a macroscopic perspective to observe the dynamic behavior of the system.

#### Evolutionary trajectory under Condition 2

**Fig 3:**
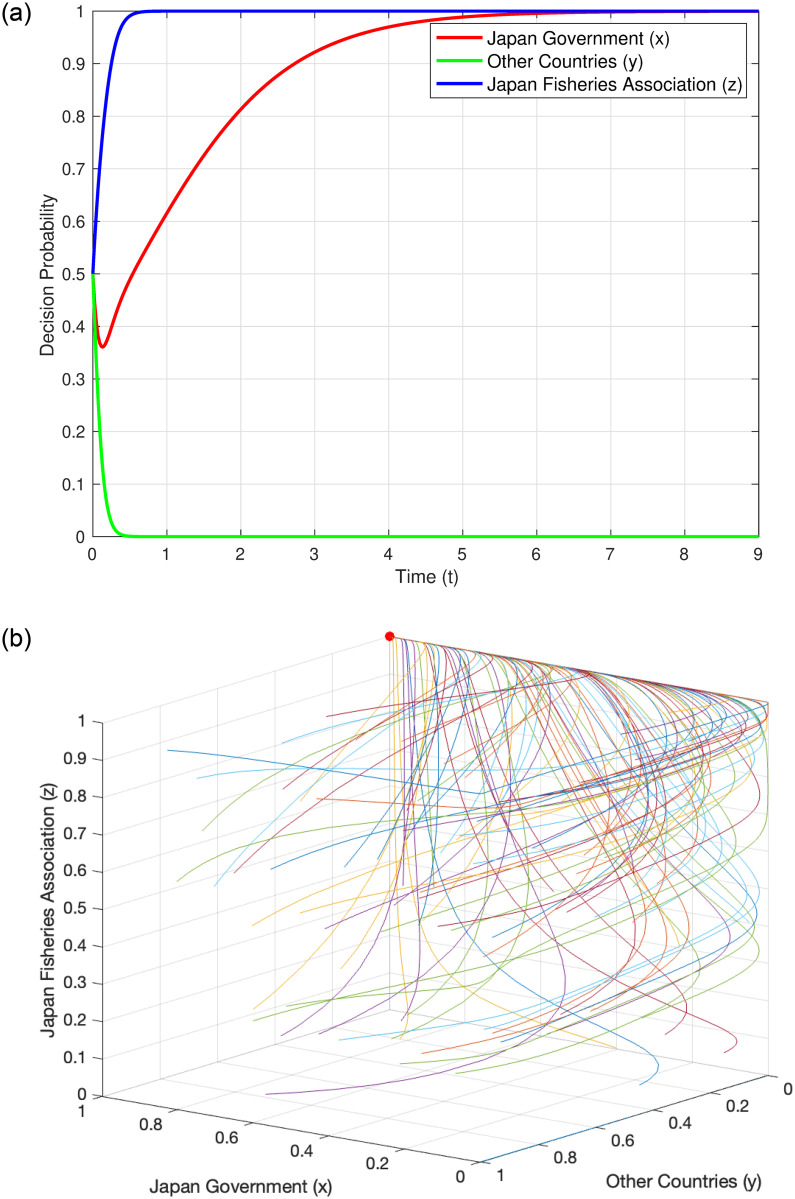


When the system satisfies Condition 2, i.e., *C*_*SJ*_ > (*C*_*LF*_ + *C*_*MJ*_ + *C*_*DJ*_) and *B*_*SP*_ + *C*_*LC*_ < *C*_*SC*_, we refer to the parameters of Condition 3 in Table 4 for analysis. This condition implies that the cost for Japan to store nuclear wastewater has exceeded the costs of discharging into the sea, marine monitoring fees, and litigation compensation to the Japanese domestic Fisheries Association; the potential benefits and litigation compensation obtained by other countries through the introduction of Japanese seafood substitutes are less than the additional costs of developing their own seafood. Under these circumstances, Japan is more likely to choose the “discharge” strategy. This decision involves considerations from multiple aspects, including economic costs, environmental impacts, social responsibility, and public opinion. The results of numerical simulation further confirm this viewpoint. The simulation results show that under this condition, the stable point of the system is (1, 0, 1). Fig 3a displays the evolution of the probability of each decision-maker over time, clearly showing how each entity gradually tends towards this stable point over time. Fig 3b illustrates the overall evolutionary trajectory under Condition 2, providing us with a macroscopic perspective to observe the dynamic behavior of the system.

#### Evolutionary trajectory under Condition 3

**Fig 4:**
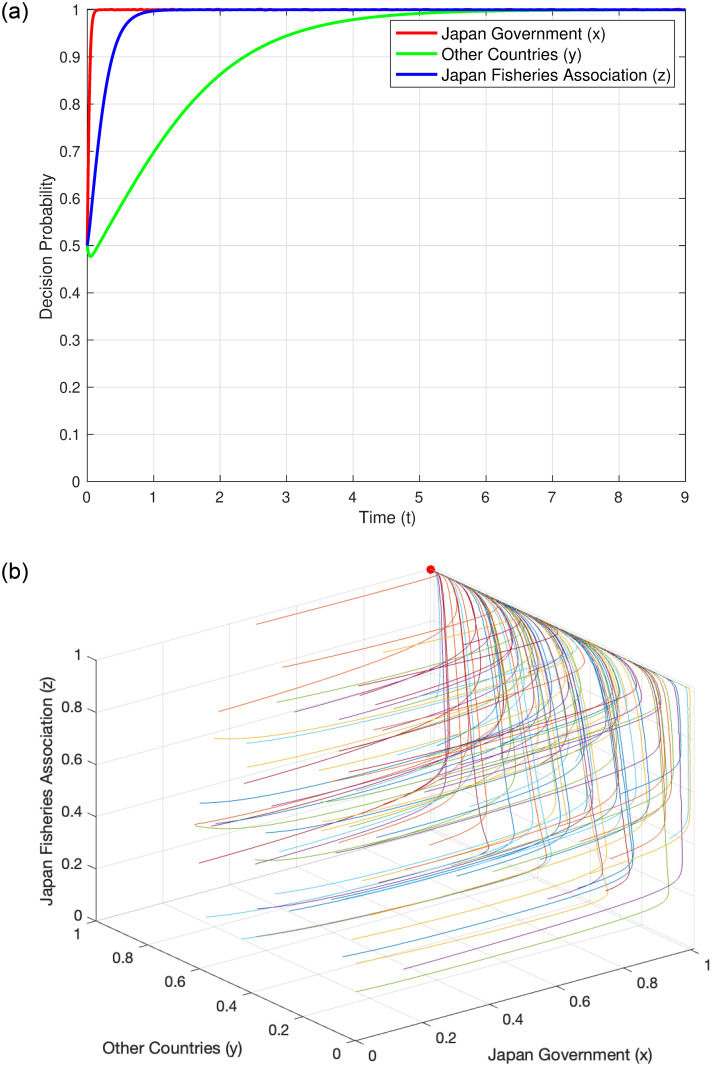


When the system satisfies Condition 3, i.e., C_SJ_ > (C_DJ_ + C_HJ_ + C_LC_ + C_LF_ + C_MJ_ + I_J_ + T_RJ_) and C_SC_ < C_LC_ + B_SP_, we refer to the parameters of Condition 3 in Table 4 for analysis. The cost for Japan to store nuclear wastewater has already exceeded the costs of discharging into the sea, marine monitoring fees, loss of international image, reduction in export taxes, and litigation compensation to the Japanese domestic Fisheries Association and other countries; the additional cost for other countries to develop their own seafood is less than the potential benefits and litigation compensation obtained by introducing Japanese seafood substitutes. Under these circumstances, Japan is more likely to choose the “discharge” strategy. The results of numerical simulation further confirm this viewpoint. The simulation results show that under this condition, the stable point of the system is (1, 0, 1). Fig 4a displays the evolution of the probability of each decision-maker over time, clearly showing how each entity gradually tends towards this stable point over time. Fig 4b illustrates the overall evolutionary trajectory under Condition 3, providing us with a macroscopic perspective to observe the dynamic behavior of the system.

### Impact of key parameters on evolutionary trajectories

Our goal is for Japan to ultimately choose the strategy to stop discharging into the sea, i.e., Condition 1. To delve deeper into the key parameters under this condition, such as the cost of Japan discharging into the sea (*C*_*DJ*_), the cost of Japan storing nuclear wastewater (*C*_*SJ*_), litigation compensation from other countries (*C*_*LC*_), litigation compensation from the Fisheries Association (*C*_*LF*_), aid received by Japan from other countries (*C*_*HJ*_), and the initial strategy choice probabilities of Japan, other countries, and the Japanese Fisheries Association (*x*_0_, *y*_0_, *z*_0_) on the Evolutionarily Stable Strategy (ESS) of the three parties, this paper has conducted a series of numerical simulations under Condition 1.

Under these settings, we explore the impact of these parameters on the evolution results and trajectories of the discharge strategy of the Japanese government, the sanction strategy of other countries, and the opposition strategy of the Japanese Fisheries Association, with specific parameter values as shown in Table 5. Our simulation results reveal how these key parameters influence the strategy choices and evolutionary dynamics of each party in the game, providing in-depth insights for our understanding of the formulation and implementation of international environmental policies.

**Table 5:** Initial parameter settings under Condition 1.

Stable point	*I*_*J*_	*C*_*LC*_	*T*_*RJ*_	*C*_*HJ*_	*C*_*LF*_	*C*_*DJ*_	*C*_*MJ*_	*C*_*SJ*_	*C*_*IF*_	*B*_*SP*_	*C*_*SC*_
*γ*_4_(0, 0, 1)^*T*^	20	8	5	10	30	3	6	30	1	1	30

#### Cost of Japan’s discharge into the sea *C*_*DJ*_

**Fig 5:**
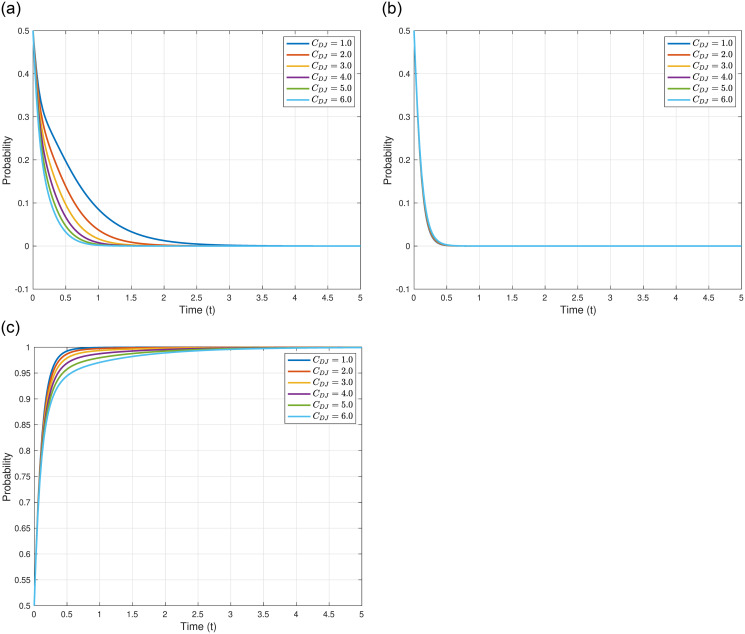


We explore the cost of Japan’s discharge into the sea, denoted as *C*_*DJ*_, on the evolutionary outcomes and trajectories of the three game entities. We assume *C*_*DJ*_ = 1.0, 2.0, 3.0, 4.0, 5.0, and 6.0, while maintaining other parameters as shown in Table 5. The specific impacts of varying *C*_*DJ*_ are illustrated in Fig 5. As illustrated in Fig 5a, with the increase in *C*_*DJ*_, the evolutionary stable point of the Japanese government, *γ*_4_(0, 0, 1)^*T*^, remains constant, but the evolutionary speed rapidly accelerates, meaning the Japanese government reaches the equilibrium point more swiftly. This might imply that higher discharge costs prompt the Japanese government to quickly solidify its strategy to mitigate additional costs induced by uncertainties. As depicted in Fig 5b, with the increase in *C*_*DJ*_, the evolutionary stable point of other countries, *γ*_4_(0, 0, 1)^*T*^, also remains constant, and the evolutionary speed does not significantly change. This might be attributed to the decision-making of other countries being predominantly influenced by their domestic factors and international political elements, having little association with Japan’s discharge costs. As shown in Fig 5c, with the increase in *C*_*DJ*_, the evolutionary stable point of the Japanese Fisheries Association, *γ*_4_(0, 0, 1)^*T*^, likewise remains constant, but the evolutionary speed decelerates, meaning the Japanese Fisheries Association reaches the equilibrium point more slowly. This might be because the Japanese Fisheries Association requires more time to assess and adapt to higher discharge costs and the potential impacts of these costs on fisheries and related industries.

In conclusion, higher costs of processing nuclear wastewater not only augment the probability of Japan adopting the discharge strategy but also expedite its evolutionary speed. The evolutionary speed and strategy selection of other countries and the Japanese Fisheries Association, on the other hand, are more influenced by their respective internal and external factors.

#### Cost of storing nuclear wastewater in Japan *C*_*SJ*_

**Fig 6:**
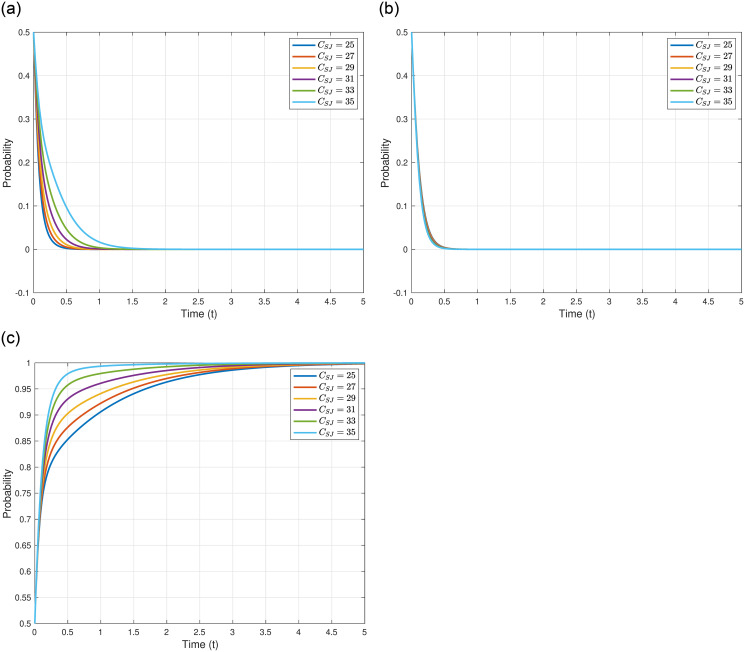


We explore the cost of storing nuclear wastewater in Japan, denoted as *C*_*SJ*_, on the evolutionary outcomes and trajectories of the three game entities. We assume *C*_*SJ*_ = 25, 27, 29, 31, 33, and 35, while maintaining other parameters as shown in Table 5. The specific impacts of varying *C*_*SJ*_ are illustrated in Fig 6. As *C*_*SJ*_ increases, the evolutionary stable point of the Japanese government, *γ*_4_(0, 0, 1)^*T*^, remains constant, but the evolutionary speed decreases, implying that the Japanese government requires more time to reach the equilibrium point. This may suggest that higher costs of storing nuclear wastewater necessitate a longer deliberation period for the Japanese government to formulate its strategy, aiming to mitigate the additional costs brought about by uncertainties. As depicted in Fig 6b, with the increase in *C*_*SJ*_, the evolutionary stable point of other countries, *γ*_4_(0, 0, 1)^*T*^, also remains unchanged, and the evolutionary speed is essentially constant. This could be attributed to the fact that the decisions of other countries are predominantly influenced by their domestic considerations and international political factors, having little correlation with Japan’s cost of discharging into the sea. As shown in Fig 6c, with the increase in *C*_*SJ*_, the evolutionary stable point of the Japanese Fisheries Association, *γ*_4_(0, 0, 1)^*T*^, also remains constant, but the evolutionary speed consistently accelerates, meaning the Japanese Fisheries Association reaches the equilibrium point more rapidly. This indicates that the Japanese Fisheries Association places a higher emphasis on environmental issues and desires the Japanese government to adopt stricter environmental protection measures promptly.

In conclusion, the varying costs of storing nuclear wastewater in Japan not only influence the strategic deliberation and evolutionary pace of the Japanese government but also reflect the environmental priorities and expectations of the Japanese Fisheries Association, while other countries remain relatively unaffected, maintaining their strategic stances based on their internal and international considerations.

#### Litigation compensation to other countries *C*_*LC*_

**Fig 7:**
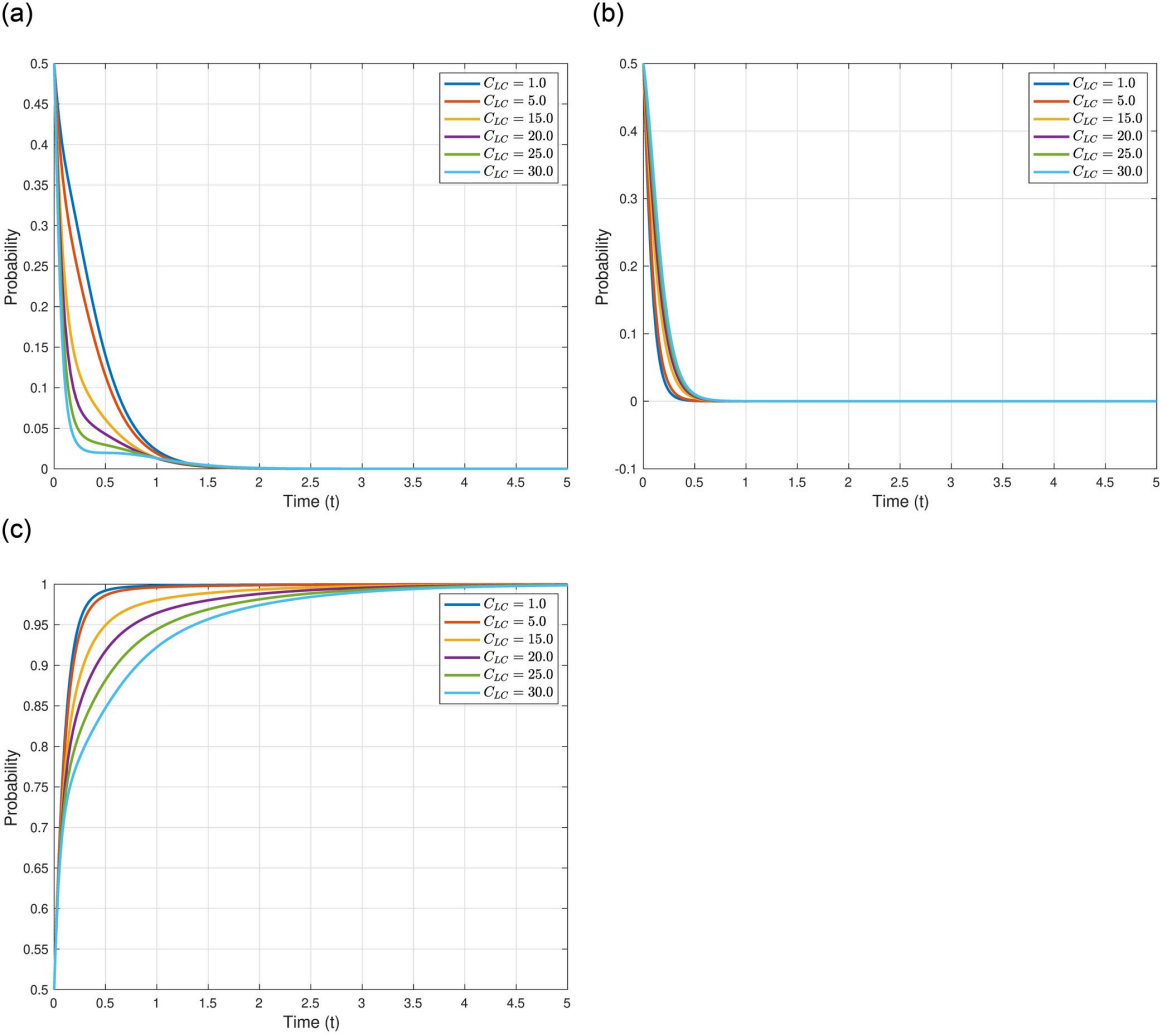


We analyze the impact of litigation compensation to other countries, denoted as *C*_*LC*_, on the evolutionary outcomes and trajectories of the three game entities. We assume *C*_*LC*_ = 1.0, 5.0, 15.0, 20.0, 25.0, and 30.0, while keeping other parameters constant as shown in Table 5. The specific impacts of varying *C*_*LC*_ are illustrated in Fig 7. Fig 7a demonstrates that as *C*_*LC*_ increases, the evolutionary stable point of the Japanese government, *γ*_4_(0, 0, 1)^*T*^, remains constant, but the evolutionary speed slows down, indicating that the Japanese government takes a longer time to reach the equilibrium point. This could signify that higher litigation costs from other countries impose greater economic pressure and uncertainty on Japan, necessitating a more cautious and deliberate approach in strategy formulation. As depicted in Fig 7b, with the increase in *C*_*LC*_, the evolutionary stable point of other countries, *γ*_4_(0, 0, 1)^*T*^, also remains constant, and the evolutionary speed gradually decelerates. This could be interpreted as other countries facing an increased number of legal disputes and litigations, requiring more resources and time to address these legal matters, thereby inducing greater economic pressure and uncertainty on their own economies. Fig 7c illustrates that with the increase in *C*_*LC*_, the evolutionary stable point of the Japanese Fisheries Association, *γ*_4_(0, 0, 1)^*T*^, also remains constant, but the evolutionary speed progressively slows down, meaning the Japanese Fisheries Association reaches the equilibrium point more slowly. This could be attributed to the Japanese Fisheries Association needing more time to assess and adapt to the potential impacts of higher litigation compensations paid by the Japanese government to other countries on the fisheries and related industries in Japan.

In conclusion, the varying levels of litigation compensation to other countries not only influence the strategic deliberation and evolutionary pace of the Japanese government and other countries but also reflect the adaptive responses and strategic considerations of the Japanese Fisheries Association in the face of increased economic pressures and uncertainties.

#### Litigation compensation to fisheries association *C*_*LF*_

**Fig 8:**
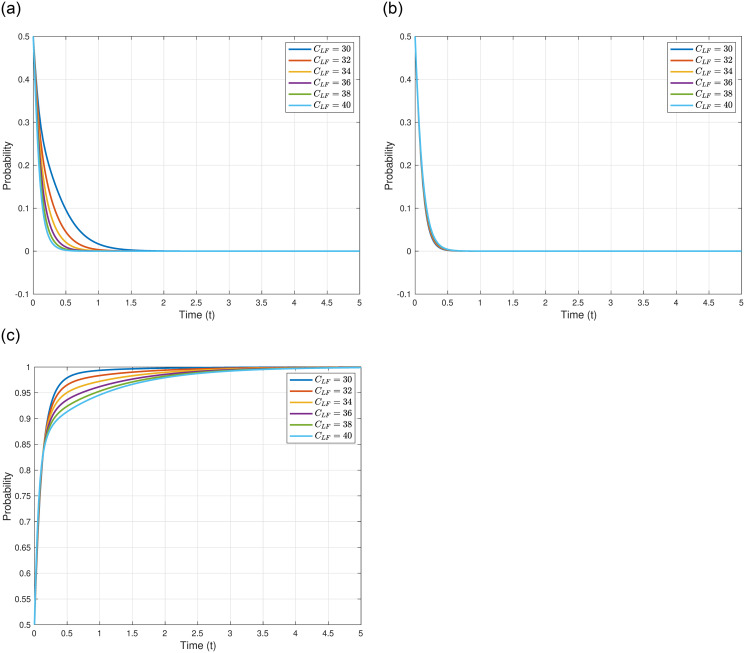


We analyze the impact of litigation compensation to the Fisheries Association, denoted as *C*_*LF*_, on the evolutionary outcomes and trajectories of the three game entities. We assume *C*_*LF*_ = 30, 32, 34, 36, 38, and 40, while keeping other parameters constant as shown in Table 5. The specific impacts of varying *C*_*LF*_ are illustrated in Fig 8. Fig 8a demonstrates that as *C*_*LF*_ increases, the evolutionary stable point of the Japanese government, *γ*_4_(0, 0, 1)^*T*^, remains constant, but the evolutionary speed rapidly accelerates, meaning the Japanese government reaches the equilibrium point more swiftly. This could signify that higher litigation costs to the Fisheries Association prompt the Japanese government to quickly solidify its strategy to mitigate additional costs induced by uncertainties, emphasizing the government’s strategic adaptability in response to internal compensatory obligations. As depicted in Fig 8b, with the increase in *C*_*LF*_, the evolutionary stable point of other countries, *γ*_4_(0, 0, 1)^*T*^, also remains constant, and the evolutionary speed does not significantly change. This could be interpreted as other countries’ decision-making being predominantly influenced by their domestic factors and international political elements, having little association with the compensations paid by the Japanese government to its domestic Fisheries Association, reflecting the relative independence of international actors from Japan’s internal compensatory dynamics. Fig 8c illustrates that with the increase in *C*_*LF*_, the evolutionary stable point of the Japanese Fisheries Association, *γ*_4_(0, 0, 1)^*T*^, also remains constant, but the evolutionary speed progressively slows down, meaning the Japanese Fisheries Association reaches the equilibrium point more slowly. This could be attributed to the Japanese Fisheries Association needing more time to assess and adapt to the potential impacts of receiving higher litigation compensations. Despite the potential influx of compensatory funds, the association must weigh the long-term considerations for the fishermen and the future development of fisheries livelihoods, highlighting the intricate balance between immediate financial relief and long-term sustainable development.

In conclusion, the varying levels of litigation compensation to the Fisheries Association not only influence the strategic deliberation and evolutionary pace of the Japanese government and the Japanese Fisheries Association but also underscore the nuanced interplay between economic compensations and sustainable strategic considerations in the context of environmental disputes.

#### Aid received by Japan from other countries *C*_*HJ*_

**Fig 9:**
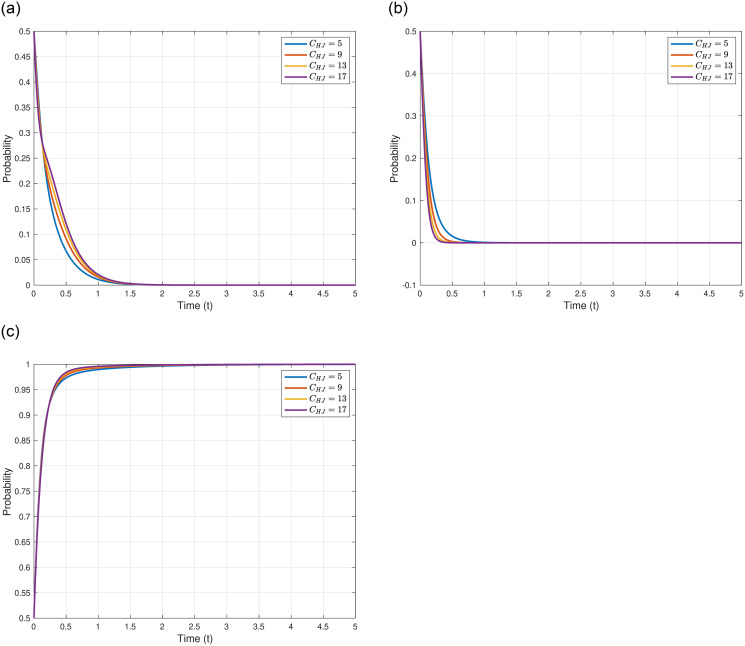


We explore the implications of the aid received by Japan from other countries, denoted as *C*_*HJ*_, on the evolutionary outcomes and trajectories of the three game entities. We assume *C*_*HJ*_ = 5, 9, 13, and 17, while maintaining other parameters as shown in Table 5. The specific impacts of varying *C*_*HJ*_ are illustrated in Fig 9. Fig 9a demonstrates that as *C*_*HJ*_ increases, the evolutionary stable point of the Japanese government, *γ*_4_(0, 0, 1)^*T*^, remains constant, but the evolutionary speed progressively decelerates, meaning the Japanese government reaches the equilibrium point more slowly. This suggests that the aid from other countries may induce the Japanese government to deliberate more meticulously on its strategies, with the slower evolutionary speed aimed at mitigating additional costs induced by uncertainties. Enhanced international aid from other countries acts as a deterrent to Japan’s discharge strategy and prolongs the time to reach the evolutionary stable point, serving as an incentivizing measure for Japan to refrain from discharging. As depicted in Fig 9b, with the increase in *C*_*HJ*_, the evolutionary stable point of other countries, *γ*_4_(0, 0, 1)^*T*^, also remains constant, but the evolutionary speed accelerates. This could be interpreted as the larger the international aid provided by other countries, the slower the progression of the crisis, and when improvements are observed, other countries also accelerate their evolution, reflecting the adaptive nature of international actors in response to the evolving crisis dynamics. Fig 9c illustrates that with the increase in *C*_*HJ*_, the evolutionary stable point of the Japanese Fisheries Association, *γ*_4_(0, 0, 1)^*T*^, also remains constant, but the evolutionary speed accelerates, meaning the Japanese Fisheries Association reaches the equilibrium point more swiftly. This could be attributed to the Japanese Fisheries Association perceiving the aid from other countries as an incentive that could potentially foster the development of Japanese fisheries and secure the income of fishermen, highlighting the perceived positive impact of international aid on domestic industries.

In conclusion, the varying levels of international aid received by Japan not only influence the strategic deliberation and evolutionary pace of the involved entities but also underscore the intricate interplay between international cooperation and strategic environmental decision-making in the context of international environmental disputes.

#### Initial strategy selection probabilities of Japan, other countries, and the japanese fisheries Association: *x*_0_, *y*_0_, *z*_0_

**Fig 10:**
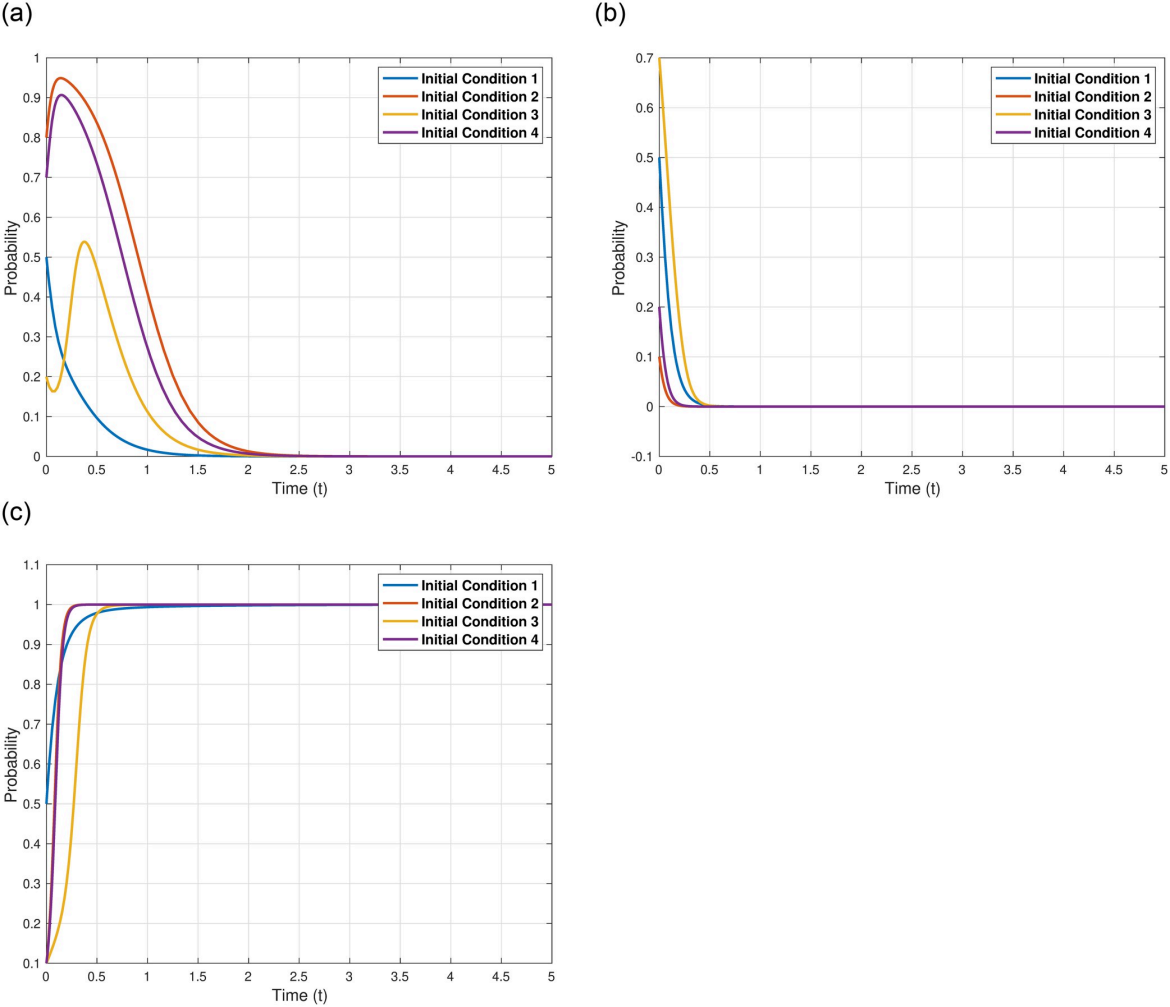


Assuming (*x*_0_, *y*_0_, *z*_0_) = (0.5, 0.5, 0.5), (0.8, 0.1, 0.1), (0.2, 0.7, 0.1), (0.7, 0.2, 0.1), while maintaining other parameters as shown in Table 5, we can observe the evolutionary outcomes and trajectories of the three game entities under varying initial strategy selection probabilities, as depicted in Fig 10. From the figure, it is evident that when initial strategy selection probabilities vary, the evolutionary stable point *γ*_4_(0, 0, 1)^*T*^ remains constant, indicating that it does not affect the evolutionary outcomes but does influence the evolutionary trajectories. For Japan, a stronger initial strategy selection probability, *x*_0_, will delay Japan’s discharge strategy and decelerate its evolutionary speed, as illustrated in Fig 10a. For other countries, the evolutionary stable point *γ*_4_(0, 0, 1)^*T*^ also remains constant when initial strategy selection probabilities vary, signifying that it does not impact the evolutionary outcomes but does have certain effects on the evolutionary trajectories. A stronger initial strategy selection probability, *y*_0_, will delay the opposition strategy of the Fisheries Association and slow down its evolutionary speed, as shown in Fig 10b. Lastly, for the Fisheries Association, the evolutionary stable point *γ*_4_(0, 0, 1)^*T*^ still remains constant when initial strategy selection probabilities change, suggesting that it does not alter the evolutionary outcomes but does affect the evolutionary trajectories. A stronger initial strategy selection probability, *z*_0_, will delay the opposition strategy of the Fisheries Association and decelerate its evolutionary speed, as depicted in Fig 10c.

## Discussion and conclusion

This study employed evolutionary game theory to explore the strategic interactions and evolutionary dynamics among Japan, other countries, and the Japanese Fisheries Association in the context of nuclear wastewater discharge. The need to understand these potential decision-making processes under varying environmental and political pressures is critical, and our research aimed to address this by delineating three evolutionarily stable strategies (ESSs) and their corresponding stability conditions. These findings reveal how different scenarios could potentially stabilize based on the strategic decisions of the involved parties. Additionally, the study highlighted the profound impact of several parameters, including Japan’s international image and economic repercussions such as litigation and export tax reductions, alongside environmental costs on the strategic behaviors of the stakeholders.

The existing literature often focuses on isolated aspects of environmental policy or employs static models that do not account for dynamic strategic interactions. By integrating evolutionary game theory, our study offers a dynamic perspective that encapsulates the complex interplay of economic, environmental, and social factors influencing policy decisions related to nuclear wastewater management. This provides a novel framework for predicting and analyzing the outcomes of such contentious environmental issues, enriching the theoretical foundations of environmental policy studies and offering a new lens through which to view the strategic decisions of countries and organizations dealing with global environmental crises.

The findings suggest that policymakers need to consider a wider array of factors, including potential international backlash and internal economic pressures, when deciding on environmental policies. We recommend that Japan reassess its approach to nuclear wastewater management, considering not only the economic but also the long-term environmental and social impacts. Furthermore, other countries and the Fisheries Association should strengthen their collaborative efforts to apply pressure and advocate for sustainable environmental practices, ensuring that their strategies are informed by both scientific research and public opinion.

In conclusion, our research illuminates the complexities involved in managing environmental crises through the lens of evolutionary game theory, providing critical insights into the potential evolutionary trajectories of policy decisions regarding nuclear wastewater discharge in Japan. The identified ESSs and the significant role of key parameters offer policymakers and stakeholders a grounded basis for reevaluating their strategies to ensure they align with both domestic needs and international expectations. This framework not only aids in understanding the current situation but also assists in forecasting and preparing for future challenges in environmental management. Our recommendations aim to foster more informed, responsible, and cooperative environmental governance, essential for addressing the multifaceted challenges posed by nuclear wastewater and other similar global environmental issues.

## Appendix

### Jacobian matrices for different points

$$\begin{array}{c} J\left(0,0,0\right)=[\begin{array}{ccc} C_{\text{SJ}}-C_{\text{MJ}}-C_{\text{DJ}}, & 0, & 0\\ 0, & -C_{\text{HJ}}, & 0\\ 0, & 0, & C_{\text{IF}} \end{array}] \end{array}$$ (26)

$$\begin{array}{c} J\left(0,0,1\right)=[\begin{array}{ccc} C_{\text{SJ}}-C_{\text{LF}}-C_{\text{MJ}}-C_{\text{DJ}}, & 0, & 0\\ 0, & -C_{\text{HJ}}, & 0\\ 0, & 0, & -C_{\text{IF}} \end{array}] \end{array}$$ (27)

$$\begin{array}{c} J\left(0,1,0\right)=[\begin{array}{ccc} C_{\text{SJ}}-C_{\text{HJ}}-C_{\text{LC}}-C_{\text{MJ}}-C_{\text{DJ}}-I_{J}-T_{\text{RJ}}, & 0, & 0\\ 0, & C_{\text{HJ}}, & 0\\ 0, & 0, & C_{\text{IF}} \end{array}] \end{array}$$ (28)

$$\begin{array}{c} J\left(0,1,1\right)=[\begin{array}{ccc} C_{\text{SJ}}-C_{\text{HJ}}-C_{\text{LC}}-C_{\text{LF}}-C_{\text{MJ}}-C_{\text{DJ}}-I_{J}-T_{\text{RJ}}, & 0, & 0\\ 0, & C_{\text{HJ}}, & 0\\ 0, & 0, & -C_{\text{IF}} \end{array}] \end{array}$$ (29)

$$\begin{array}{c} J\left(1,0,0\right)=[\begin{array}{ccc} C_{\text{DJ}}+C_{\text{MJ}}-C_{\text{SJ}}, & 0, & 0\\ 0, & \;B_{\text{SP}}+C_{\text{LC}}-C_{\text{SC}}, & 0\\ 0, & 0, & C_{\text{LF}}+C_{\text{IF}} \end{array}] \end{array}$$ (30)

$$\begin{array}{c} J\left(1,0,1\right)=[\begin{array}{ccc} C_{\text{DJ}}+C_{\text{LF}}+C_{\text{MJ}}-C_{\text{SJ}}, & 0, & 0\\ 0, & \;B_{\text{SP}}+C_{\text{LC}}-C_{\text{SC}}, & 0\\ 0, & 0, & -C_{\text{IF}}-C_{\text{LF}} \end{array}] \end{array}$$ (31)

$$\begin{array}{c} J\left(1,1,0\right)=[\begin{array}{ccc} C_{\text{DJ}}+C_{\text{HJ}}+C_{\text{LC}}+C_{\text{MJ}}-C_{\text{SJ}}+I_{J}+T_{\text{RJ}}, & 0, & 0\\ 0, & C_{\text{SC}}-C_{\text{LC}}-B_{\text{SP}} & 0\\ 0, & 0, & C_{\text{LF}}+C_{\text{IF}} \end{array}] \end{array}$$ (32)

$$\begin{array}{c} J\left(1,1,1\right)=[\begin{array}{ccc} C_{\text{DJ}}+C_{\text{HJ}}+C_{\text{LC}}+C_{\text{LF}}+C_{\text{MJ}}-C_{\text{SJ}}+I_{J}+T_{\text{RJ}}, & 0, & 0\\ 0, & C_{\text{SC}}-C_{\text{LC}}-B_{\text{SP}}, & 0\\ 0, & 0, & -C_{\text{IF}}-C_{\text{LF}} \end{array}] \end{array}$$ (33)

### Eigenvalues for different points

$$\begin{array}{c} \gamma_{1}\left(0,0,0\right)=(\begin{array}{c} C_{\text{SJ}}-C_{\text{MJ}}-C_{\text{DJ}}\\ -C_{\text{HJ}}\\ C_{\text{IF}} \end{array}) \end{array}$$ (34)

$$\begin{array}{c} \gamma_{2}\left(1,0,0\right)=(\begin{array}{c} C_{\text{DJ}}+C_{\text{MJ}}-C_{\text{SJ}}\\ B_{\text{SP}}+C_{\text{LC}}-C_{\text{SC}}\\ C_{\text{LF}}+C_{\text{IF}} \end{array}) \end{array}$$ (35)

$$\begin{array}{c} \gamma_{3}\left(0,1,0\right)=(\begin{array}{c} C_{\text{SJ}}-C_{\text{HJ}}-C_{\text{LC}}-C_{\text{MJ}}-C_{\text{DJ}}-I_{J}-T_{\text{RJ}}\\ C_{\text{HJ}}\\ C_{\text{IF}} \end{array}) \end{array}$$ (36)

$$\begin{array}{c} \gamma_{4}\left(0,0,1\right)=(\begin{array}{c} C_{\text{SJ}}-C_{\text{LF}}-C_{\text{MJ}}-C_{\text{DJ}}\\ -C_{\text{HJ}}\\ -C_{\text{IF}} \end{array}) \end{array}$$ (37)

$$\begin{array}{c} \gamma_{5}\left(1,1,0\right)=(\begin{array}{c} C_{\text{DJ}}+C_{\text{HJ}}+C_{\text{LC}}+C_{\text{MJ}}-C_{\text{SJ}}+I_{J}+T_{\text{RJ}}\\ C_{\text{SC}}-C_{\text{LC}}-B_{\text{SP}}\\ C_{\text{LF}}+C_{\text{IF}} \end{array}) \end{array}$$ (38)

$$\begin{array}{c} \gamma_{6}\left(1,0,1\right)=(\begin{array}{c} C_{\text{DJ}}+C_{\text{LF}}+C_{\text{MJ}}-C_{\text{SJ}}\\ B_{\text{SP}}+C_{\text{LC}}-C_{\text{SC}}\\ -C_{\text{IF}}-C_{\text{LF}} \end{array}) \end{array}$$ (39)

$$\begin{array}{c} \gamma_{7}\left(1,0,1\right)=(\begin{array}{c} C_{\text{SJ}}-C_{\text{HJ}}-C_{\text{LC}}-C_{\text{LF}}-C_{\text{MJ}}-C_{\text{DJ}}-I_{J}-T_{\text{RJ}}\\ C_{\text{HJ}}\\ -C_{\text{IF}} \end{array}) \end{array}$$ (40)

$$\begin{array}{c} \gamma_{8}\left(1,1,1\right)=(\begin{array}{c} C_{\text{DJ}}+C_{\text{HJ}}+C_{\text{LC}}+C_{\text{LF}}+C_{\text{MJ}}-C_{\text{SJ}}+I_{J}+T_{R}J\\ C_{\text{SC}}-C_{\text{LC}}-B_{\text{SP}}\\ -C_{\text{IF}}-C_{\text{LF}} \end{array}) \end{array}$$ (41)

## Supporting information

S1 File (DOCX)
