# Supplementary material for: An evolutionary game theory analysis on the environmental impact of discharging Fukushima’s nuclear wastewater: International stakeholders and strategic dynamics
Source: PLoS One. 2025 Jan 27;20(1):e0317419. doi: 10.1371/journal.pone.0317419 (PMC11771948; doi:10.1371/journal.pone.0317419)
Supplement: S1 File — (DOCX) [file pone.0317419.s001.docx]

To enhance the openness and facilitate academic communication, all programs and data associated with this paper are publicly available. The complete datasets and codes required to replicate the findings of this study can be accessed via the following links: [Figshare](https://doi.org/10.6084/m9.figshare.26308606.v1) and [GitHub](https://github.com/MingyangLi7278/DATA-in-Article). We ensure full reproducibility of the results presented in this article.
